# Prospective Associations Between Social Connectedness and Mental Health. Evidence From a Longitudinal Survey and Health Insurance Claims Data

**DOI:** 10.3389/ijph.2022.1604710

**Published:** 2022-06-09

**Authors:** Dorota Weziak-Bialowolska, Piotr Bialowolski, Matthew T. Lee, Ying Chen, Tyler J. VanderWeele, Eileen McNeely

**Affiliations:** ^1^ Department of Environmental Health, School of Public Health, Harvard University, Boston, MA, United States; ^2^ Institute for Quantitative Social Science, Harvard University, Cambridge, MA, United States; ^3^ Center for Evaluation and Analysis of Public Policies, Faculty of Philosophy, Jagiellonian University, Kraków, Poland; ^4^ Department of Economics, Kozminski University, Warsaw, Poland; ^5^ Department of Epidemiology, School of Public Health, Harvard University, Boston, MA, United States

**Keywords:** anxiety, loneliness, mental health, depression, health insurance data, social connectedness

## Abstract

**Objectives:** Evidence on social stimuli associated with mental health is based mostly on self-reported health measures. We aimed to examine prospective associations between social connectedness and clinical diagnosis of depression and of anxiety.

**Methods:** Longitudinal observational data merged with health insurance data comprising medical information on diagnosis of depression and anxiety were used. 1,209 randomly sampled employees of a US employer provided data for the analysis. Robust Poisson regression models were used. Multiple imputation was conducted to handle missing data on covariates.

**Results:** Better social connectedness was associated with lower risks of subsequently diagnosed depression and anxiety, over a one-year follow-up period. Reports of feeling lonely were associated with increased risks of depression and anxiety. Association between community-related social connectedness and subsequent diagnosis of depression, but not of anxiety, was found. The associations were independent of demographics, socioeconomic status, lifestyle, and work characteristics. They were also robust to unmeasured confounding, missing data patterns, and prior health conditions.

**Conclusion:** Social connectedness may be an important factor for reducing risks of depression and anxiety. Loneliness should be perceived as a risk factor for depression and anxiety.

## Introduction

Growing evidence in literature shows that positive social factors are effective in improving mental health and emotional well-being as well as in reducing risks of ill-being. Various conceptualizations of these social factors have been used to establish the link.

A number of rigorous longitudinal studies have identified positive temporal associations between advantageous, individual and relational social stimuli and health. For example, better social integration and cohesion have been shown to contribute to longevity and healthy life expectancy, reduced risk of all-cause mortality and mortality from cardiovascular disease, lower risks of hypertension, pulmonary disease, and abdominal obesity, better general metabolic health, and improved measures of physical and mental health [1–4]. Social engagement has been found to be associated with lower levels of inflammation [5, 6]. Membership in social groups, close relationships, and friendship have been evidenced to protect against depression, alleviate depression symptoms and prevent depression relapse [7] as well as positively contribute to sense of purpose in life [8]. Experimental evidence corroborated the important role of social support in reducing risks of feeling lonely, depressed and incidence of psychosis [9]. Meta-analytical evidence based on 12,778 estimates from 470 studies on the links between social capital and a number of health outcomes indicated positive associations in this respect [10].

Disadvantageous social factors, such as loneliness and feeling of alienation, have been associated with lower purpose in life [8], depression [11–14], anxiety [11, 15], suicidal ideation [11, 13, 14, 16], suicidal behavior [13] and worse regulation of inflammation [6]. Finally, the presence of a vicious cycle between feelings of loneliness and social anxiety and depression has been also reported among adolescents and older adults [12, 15].

Although previous studies have substantially advanced our understanding of the importance of social factors for health and longevity, they were subject to some limitations. First, a recent meta-analysis reported that although social capital is significantly associated with a number of health outcomes, the effect sizes are consistently very small [10]. This cast some doubt on the effectiveness of various mental health promoting initiatives in which social capital and social well-being are used as intervention factors. Second, previous studies focused on the impacts of social factors on self-reported health outcomes, and thus provided limited evidence on their associations with objectively measured health conditions. Additionally, it has been shown that self-reported and clinical diagnosis of a disease may be discrepant [17, 18], with only a fair level of coherence between those measures for depression and anxiety [19, 20]. Third, many studies on the importance of social factors (especially on social cohesion) for health and well-being were conducted on older adults and usually omitted work-related factors despite their well-known contribution to mental health [21, 22].

Therefore, this study aims to examine the prospective associations between social connectedness and two mental health outcomes: clinical diagnosis of depression and clinical diagnosis of anxiety. We focus on working adults and control for the role of work-related covariates for mental health as their importance for health was indicated by prior research (e.g., [23, 24]. Inclusion of work-related factors strengthens our results and provides additional evidence on their robustness. This study also benefits from prior work on the conceptualization and measurement of social connectedness in a working setting [25]. Specifically, we utilized a well-validated scale of social connectedness [25]. We hypothesize that social connectedness will be negatively associated with the risk of the examined mental health outcomes. To test this hypothesis, we used both longitudinal survey data and data on medical diagnoses derived from health insurance claims records.

## Methods

### Data

The data were gathered in June 2018 (wave 1) and July 2019 (wave 2) as part of a project designed to assess well-being in life and well-being at work among employees of a large, national company based in the United States. In the first wave (June 2018) 2,370 individuals participated. Only first-wave participants were invited to the second wave (July 2019). 1,209 respondents took part in the second data collection (the retention rate between waves was 51.2%). All present employees aged 18 and above were entitled to participate in the survey. Participation was voluntary and confidential. Informed written consent was acquired from all participants. All protocols for recruitment and participation were reviewed and approved by the Harvard Longwood Campus Institutional Review Board. More details on the sample and the study can be found in [26–28].

The analytic sample was drawn from participants who participated in both waves of the data collection (N = 1,209; missing observations accounted for 1.15% of total number of survey data; there were no missing observations in the data from health insurance claims; Supplementary Table SA1). For this sample, the survey records (two waves: 2018 and 2019) were merged with the health insurance records (from 2017, 2018, and 2019). Our interest was in the diagnostic information on medical conditions included in health insurance records, following the International Classification of Diseases (ICD-10) [29]. Table 1 presents the descriptive statistics at baseline (wave 1, 2018, T = 0) [26].

**TABLE 1:** Participant characteristics at study baseline (T = 0; N = 1,209; Well-Being Survey 2018–2019 and health insurance claims data 2017–2019, United States).

Baseline characteristic	Statistic
Gender (women), %	84.45
Age—mean (SD)	43.52 (10.4)
Age, %	
≤30	11.83
31–40	29.94
41–50	28.95
>50	29.28
Race, %	
White	74.28
Black or African American	12.16
Hispanic/Latino	6.70
Asian	5.05
Other	1.81
Marital status (married), %	62.47
Education, %	
High school	7.78
Some college but no degree	22.58
Associate degree	13.96
Bachelor’s degree	34.95
Graduate school or higher	20.74
Having children under the age of 18 currently living in the household, % of yes	48.11
Being a primary caregiver for a parent or an elderly currently living in the household, % of yes	27.17
Home ownership, % of yes	72.36
Salary (USD)—mean (SD)	73,117 (34,259)
Voting in the previous elections, % of yes	82.40
Spiritual practicing, %	
At least once/week	61.15
Less than once/week	30.66
Never	8.19
Work hours, %	
≤8 h	52.34
9–10 h	35.37
11+ h	12.29
Job demand: I have too much to do at work to do a good job (0–10)—mean (SD)	3.18 (2.76)
Job control: I have a lot of freedom to decide how to do my job (0–10)—mean (SD)	7.03 (2.50)
Job meaning: I find my work meaningful (0–10)—mean (SD)	7.55 (2.10)
Job fit: At work, I am able to do what I am good at (0–10)—mean (SD)	7.63 (2.12)
Supervisor support: My supervisor supports me (0–10)—mean (SD)	8.44 (2.10)
Number of health conditions (0–37)—mean (SD)	2.02 (2.25)

Note. Adapted from [28]. CC BY-NC-ND.

The dataset will be openly available at https://doi.org/10.7910/DVN/RTB1OL at the conclusion of the 5-year funding period, which ends November 14, 2022. Until then, the data are available for scholarly research from the first author upon request.

### Measures

#### Mental Health Outcomes

We examined two mental health outcomes from medical insurance claims data: (1) clinical diagnosis of depression and (2) clinical diagnosis of anxiety. Each of these two outcomes was measured by a dichotomous variable with the value of 1, when the appropriate diagnosis was recorded in the insurance claims data and 0 otherwise.

#### Exposure: Social Connectedness

A social connectedness instrument from the Well-Being Assessment [25, 27] was applied. The prior research indicated that a single total score across seven items populating the social connectedness domain of well-being is a psychometrically sound instrument [25]. Consequently, the social connectedness score was calculated by averaging the responses across all seven items included in the domain of social connectedness.

The items of social connectedness were designed to reflect aspects of social connection and intimacy, social support and communal social well-being and included: 1) “My relationships are as satisfying as I would want them to be,” 2) “There are people who really understand me,” 3) “I am content with my friendships and relationships,” 4) “I have enough people I feel comfortable asking for help at any time,” 5) “How often do you feel lonely?” (negatively oriented), 6) “I feel connected to the broader community around me,” and 7) “People in my broader community trust and respect one another.” Respondents chose their responses on a 0–10 scale, with higher number corresponding to better social connectedness and 5 being labelled as neutral.

In the analyses, we included a domain specific score reflecting overall social connectedness. We also used each of the seven items of the social connectedness domain as an independent variable separately [these variables were applied as standardized continuous variables (mean = 0, standard deviation = 1)], as prior research showed that longitudinal associations between overall flourishing and individual items of social connectedness vs. overall social connectedness score yielded different results [30].

#### Covariates

Covariates included the following sociodemographic characteristics: participant age, gender, race, and highest educational attainment (response categories presented in Table 1). We also considered marital status, having children at home, taking care of an elderly, and salary (measured using the mid-point salary bands obtained from the human resource department of the employer). Additionally, we controlled for wealth (measured via a proxy variable: owning a house. Participants were also queried about their civic engagement [voting in the last elections] and spiritual practices. Work characteristics were identified based on the two theoretical models of job strain: 1) job control-demand model [31, 32], and 2) job demands-resources model [33]. Consequently, we controlled for six work characteristics: 1) number of work hours, 2) job demand, 3) job control, 4) job meaning, 5) job fit, and 6) supervisor support. Observations from the first wave (in the same wave as the exposure) were used, since only two waves of survey data were available.

#### Prior Values of the Outcome Variables

In each regression we controlled for prebaseline values of outcome variables, to reduce the risk of reverse causation. Adjustments were also made for the number of diagnosed chronic health conditions prior to exposure to account for previous health conditions and to further minimize possibility of reverse causality. A similar set of controls was applied in previous studies on similar topics [26].

### Statistical Analysis

Two waves of data were used to establish whether the social connectedness is associated with subsequent mental health outcomes. A robust Poisson regression was applied to estimate risk ratios for (1) diagnosed depression, and (2) diagnosed anxiety, since both outcomes were on the edge of the rare/non-rare disease threshold of 10% ([34]; see Table 2). In robust Poisson regression, a quasi-likelihood model is applied to fit the data with a binary outcome [35, 36]. This method is recommended and useful when log-binomial model does not converge—mostly due to the use of multiple control variables of a dichotomous and continuous nature as evidenced by previous literature [35–37].

**TABLE 2:** Social connectedness and mental health (N = 1,209; Well-Being Survey 2018–2019 and health insurance claims data 2017–2019, United States).

Characteristic	2017	2018	2019	*p*-value for one-sided t-test for paired observations	
				2017–2018	2018–2019
Social connectedness (0–10)	—	7.36	7.58	—	<0.001
Items of social connectedness					
My relationships are as satisfying as I would want them to be; (0–10); mean (SD)	—	7.10 (2.20)	7.46 (2.00)	—	<0.001
There are people who really understand me; (0–10); mean (SD)	—	7.88 (2.09)	8.08 (1.95)		<0.001
How often do you feel lonely?; reversed; (0–10); mean (SD)	—	2.25 (2.39)	2.39 (2.47)	—	0.012
I am content with my friendships and relationships; (0–10); mean (SD)	—	7.57 (2.09)	7.85 (1.98)	—	<0.001
I have enough people I feel comfortable asking for help at any time; (0–10); mean (SD)	—	7.69 (2.35)	7.90 (2.18)	—	<0.001
I feel connected to the broader community around me; (0–10); mean (SD)	—	6.66 (2.26)	7.04 (2.16)	—	<0.001
People in my broader community trust and respect one another; (0–10); mean (SD)	—	6.88 (2.00)	7.18 (1.94)	—	<0.001
Mental health outcomes from health insurance records					
Depression, %	9.59	10.42	12.65	0.070	<0.001
Anxiety, %	12.66	12.16	13.40	0.760	0.062

Note. “—” stands for outcome not measured; *p*-value for one-sided t-test for paired observations for changes in diagnosed depression between 2017 and 2019: *p* < 0.001; *p*-value for one-sided t-test for paired observations for changes in diagnosed anxiety between 2017 and 2019: *p*-value = 0.219.

Adapted from [26]. CC BY-NC-ND.

To estimate the risk ratio for each mental health outcome, the outcome at the follow-up wave (T = 1) was regressed on social connectedness indicator at baseline (T = 0) (or on each indicator of social connectedness), adjusting for covariates [including the prebaseline value of the outcome variable (T = −1) and prebaseline number of diagnosed health conditions to control for the history of diseases (T = −1), as well as baseline values of other controls (T = 0)]. This specification is recommended to examine prospective associations in longitudinal designs with an outcome temporarily subsequent to the exposure [38, 39].

Imputations using chained equations (with 20 sets of imputed data) [40] were applied to account for missing covariates and exposure variables (only from the survey data as there were no missing observations in the data from health insurance claims, which also meant that there were no missing observations in the outcome variables). The multiple imputation estimates were pooled using the Rubin’s rule [41].

A set of robustness analyses was conducted. First, additional analyses were conducted on a restricted sample of those without mental health diseases at baseline (Model 2; as opposed to the primary analysis based on the entire sample with the control for the outcome prior to exposure, Model 1). Second, the original analyses were recomputed using the complete case scenario to examine robustness of the results to missing data patterns (Model 3). Third, the model with limited number of control variables (the prebaseline outcome (T = −1) and the prebaseline history of diseases (T = −1), only) was also estimated to examine robustness of the results to the risk of overfitting, possibly resulting from an extensive set of controls used originally (Model 4). Fourth, different specifications of the model were applied to provide further evidence on the directionality of the prospective associations. Specifically, the cross-lagged panel model was run in which reciprocal prospective associations were assumed and four paths were estimated simultaneously (i.e., from a mental health outcome in T = 0 to social connectedness in T = 1, from social connectedness in T = 0 to a mental health outcome in T = 1, and autoregressive paths: from social connectedness in T = 0 to T = 1, and from a mental health outcome in T = 0 to T = 1; controlling for the set of covariates from the primary analysis and pre-exposure history of diseases; Model 5). Further robustness checks were also conducted and are presented in Supplementary Materials, Supplementary Analyses, Models S1, S2, S3.1–S3.3.

Secondary analyses were also run on single-item indicators of social connectedness. Each mental health outcome was regressed on each indicator of social connectedness separately to provide further insights into the differential associations between social factors and subsequent health (with a distinction between indicators of cognitive social capital and structural social capital).

Finally, a sensitivity analysis was run to examine robustness of the results to unmeasured confounding. To this end, E-values were calculated [42]. Their role is to assess the minimum strength of association on the risk ratio scale that an unmeasured confounder would need to have with both exposure and outcome to explain away their observed association, above and beyond the measured covariates.

Analyses were performed using Stata/SE 17.0.

## Results

### Characteristics of the Study Participants

In the baseline wave (T = 0), participants were 43.5 (SD = 10.4) years old, on average (Table 1; Supplementary Materials, Characteristics of Study Participants). They were mostly women (84.5%), married (62.5%), predominantly Caucasian (74.3), and with Bachelor’s degree (35.0%).

With respect to mental health in particular, in the baseline wave (T = 0) 10.4% of the participants were diagnosed with depression and 12.2% were diagnosed with anxiety (Table 2). No significant changes in anxiety were noted between the prebaseline (2017) and 2019. Prevalence of depression increased over this period. Regarding social connectedness, in the baseline wave (T = 0, 2018), participants scored 7.4 on average on a 0–10 response scale (Table 2, [26]).

### Social Connectedness and Mental Health

Social connectedness was found to be significantly associated with subsequent reduced odds of depression and anxiety (Table 3, Model 1). After adjusting for covariates, an increase by one standard deviation in the social connectedness composite score was associated with a 27% reduced risk of clinically diagnosed depression (RR = 0.730, 95% CI: 0.637, 0.837) over a one-year follow-up period. Additionally, an increase by one standard deviation in the social connectedness composite score was associated with a 18% reduced risk of clinically diagnosed anxiety (RR = 0.824, 95% CI: 0.719, 0.943) over a one-year follow-up period.

**TABLE 3:** Associations between social connectedness and subsequent mental health (Well-Being Survey 2018–2019 and health insurance claims data 2017–2019, United States)^a^.

Model specification	Depression	Anxiety
	RR 95% CI	RR 95% CI
Model 1^b^	0.730*** (0.637, 0.837)	0.824** (0.719, 0.943)
Model 2^c^	0.554*** (0.438, 0.700)	0.691** (0.550, 0.868)
Model 3^d^	0.858*** (0.794, 0.928)	0.894** (0.825, 0.969)
Model 4^e^	0.784*** (0.696, 0.882)	0.835** (0.743, 0.940)
Model 5^f^	0.669** (0.505, 0.884)	0.735* (0.576, 0.937)

a Each analysis was controlled for demographics [gender (ref. = female), age (ref. = below 30), race (ref. = White), education (ref. = high school), marital status (ref. = not married)), having children at home (ref. = no), taking care of an elderly (ref. = no)], wealth and income [home ownership (ref. = no) and salary], lifestyle [voting in the last elections (ref. = no/not registered voter) and spiritual practices (ref. = at least once/week)] and work characteristics (number of work hours (ref. = ≤8 h), supervisor support, job control, job demand and job meaning). These variables were controlled for in the baseline wave, T = 0 (in the same wave as the exposure), since only two waves of survey data were available. Additionally, in each regression an outcome prior to exposure (T = −1) as well as the number of diagnosed health conditions (ranging from 0 to 37 possible diagnosed health conditions) prior to exposure (T = −1) were used as controls.

b Model 1 is run on the full imputed analytic sample.

c Model 2 is run after excluding participants with baseline mental health condition (i.e., either depression or anxiety).

d Model 3 is run under the complete case scenario.

e Model 4 uses a limited set of baseline controls (T = 0) including only the outcome prior to exposure (T = −1) and pre-exposure number of health conditions (T = −1).

f Model 5 is run as a cross-lagged panel model using the set of controls from the primary analysis; under complete case scenario as Stata *gsem* command does not support *mi estimate* command used to account for multiple imputations; uses a logistic regression specification as *gsem* does not support robust Poisson regression. Odds ratio for the path from social connectedness to mental health outcome is presented. Full set of results is presented in the Supplementary Table SA3).

***p < 0.001, **p < 0.01, *p < 0.05; CI is confidence interval.

CI, confidence interval; RR, risk ratio.

### Robustness Analysis

Robustness analyses (Table 3, Models 2–5) corroborated results of the primary analysis. The results obtained on a limited sample of respondents who did not report the examined health outcome prior to exposure, showed even stronger associations with prior social connectedness (Table 3, Model 2). In particular, one standard deviation increase in the social connectedness score was associated with a reduction in the risk of prospective clinical diagnosis of depression by 27.0% in the entire sample and in the limited sample of individuals not diagnosed with depression prior to exposure a decrease in the risk by 45% was observed.

Next, the analyses conducted using the complete case scenario provided further evidence for the robustness of the results. The results showed that the associations were robust to the missing data pattern (Table 3, Model 3), however the effect sizes were slightly attenuated. Similarly, the analyses with the use of limited sets of controls (Table 3, Model 4) showed that the pattern of significant associations remained the same with very comparable effect sizes. Finally, the analyses run under the cross-lagged panel model specification, which assumes simultaneous reciprocal prospective associations between social connectedness and mental health outcome, showed that there was an association between social connectedness and each of the subsequent mental health outcomes examined (Table 3, model 5). Additionally, the association in the opposite direction (from mental health outcome to subsequent social connectedness) was present only for depression and not for anxiety (Supplementary Table SA2).

Results from the supplementary analyses, which tested alternative specifications (Supplementary Materials, Supplementary Analyses) provided further evidence on the robustness of prospective associations.

### Sensitivity Analysis

The sensitivity analysis conducted using E-values provided additional evidence on the robustness of the results to unmeasured confounding (Table 4). For the social connectedness composite score we found that the associations with both diagnosed depression and diagnosed anxiety were moderately robust to unmeasured confounding.

**TABLE 4:** E-values for effect measures and for CI limits for the associations between social capital and subsequent mental health (Well-Being Survey 2018–2019 and health insurance claims data 2017–2019, United States).

Social connectedness	Depression		Anxiety	
	Effect estimate^a^ ^,^ ^b^	CI limit^c^	Effect estimate^b^	CI limit^c^
Social connectedness	2.08	1.68	1.72	1.31
Items of social connectedness				
My relationships are as satisfying as I would want them to be	1.69	1.27	1.51	1.00
There are people who really understand me	1.71	1.36	1.53	1.03
How often do you feel lonely?	1.96	1.60	1.71	1.37
I am content with my friendships and relationships	1.83	1.45	1.64	1.24
I have enough people I feel comfortable asking for help at any time	1.74	1.41	1.67	1.29
I feel connected to the broader community around me	1.78	1.40	1.35	1.00
People in my broader community trust and respect one another	1.91	1.52	1.45	1.00

a See VanderWeele and Ding [42] for the formula for calculating E-values.

b The E-values for effect estimates are the minimum strength of association on the risk ratio scale that an unmeasured confounder would need to have with both the exposure and the outcome to fully explain away the observed association between the exposure and outcome, conditional on the measured covariates. For example, in the studied population an unmeasured confounder would need to be associated with both feeling lonely and depression by risk ratios of 1.96 each, above and beyond the measured covariates, to fully explain away the observed association between the two variables.

c The E-values for the limit of the 95% confidence interval (CI) closest to the null denote the minimum strength of association on the risk ratio scale that an unmeasured confounder would need to have with both the exposure and the outcome to shift the confidence interval to include the null value, conditional on the measured covariates. For example, in the studied population an unmeasured confounder would need to be associated with both feeling lonely and depression by 1.60-fold each, above and beyond the measured covariates, to shift the upper limit of the confidence interval to include the null value for the association between the two variables.

### Indicators of Social Connectedness and Mental Health

Indicators of social connectedness were found to be significantly associated with subsequent reduced odds of depression and anxiety (Table 5). After adjusting for covariates, an increase by one standard deviation in the assessment of relationships was associated with a 17% reduced risk of clinically diagnosed depression (RR = 0.833, 95% CI: 0.727, 0.954) over a one-year follow-up period. A belief of having people who really understand respondent was also prospectively associated with a 17% and a 12% reduced risk of both clinically diagnosed depression and clinically diagnosed anxiety, respectively (RR = 0.828, 95% CI: 0.736, 0.931 and RR = 0.880, 95% CI: 0.775, 0.999, respectively). Feeling lonely was prospectively associated with a 32% increased risk of diagnosed depression (RR = 1.315, 95% CI: 1.164, 1.486) and a 21% increased risk of anxiety (RR = 1.211, 95% CI: 1.078, 1.359) over a one-year follow-up period.

**TABLE 5:** Associations between items of social connectedness and subsequent mental health (Well-Being Survey 2018–2019 and health insurance claims data 2017–2019, United States)^a^.

Item of social connectedness	Depression	Anxiety
	RR 95% CI	RR 95% CI
My relationships are as satisfying as I would want them to be	0.833** (0.727, 0.954)	0.886 (0.778, 1.008)
There are people who really understand me	0.828** (0.736, 0.931)	0.880* (0.775, 0.999)
How often do you feel lonely?	1.315*** (1.164, 1.486)	1.210** (1.078, 1.359)
I am content with my friendships and relationships	0.794** (0.698, 0.904)	0.847* (0.746, 0.962)
I have enough people I feel comfortable asking for help at any time	0.818*** (0.731, 0.916)	0.840** (0.743, 0.950)
I feel connected to the broader community around me	0.809** (0.711, 0.919)	0.933 (0.818, 1.065)
People in my broader community trust and respect one another	0.773*** (0.676, 0.884)	0.904 (0.777, 1.051)

a Each analysis was controlled for demographics [gender (ref. = female), age (ref. = below 30), race (ref. = White), education (ref. = high school), marital status (ref. = not married)), having children at home (ref. = no), taking care of an elderly (ref. = no)], wealth and income [home ownership (ref. = no) and salary], lifestyle [voting in the last elections (ref. = no/not registered voter) and spiritual practices (ref. = at least once/week)] and work characteristics (number of work hours (ref. = ≤8 h), supervisor support, job control, job demand and job meaning). These variables were controlled for in the baseline wave, T = 0 (in the same wave as the exposure), since only two waves of survey data were available. Additionally, in each regression an outcome prior to exposure (T = −1) as well as the number of diagnosed health conditions (ranging from 0 to 37 diagnosed health conditions) prior to exposure (T = −1) were used as controls.

***p < 0.001, **p < 0.01, *p < 0.05; CI is confidence interval.

CI, confidence interval; RR, risk ratio.

Variables related to feeling content with one’s friendships and relations as well as having enough people to comfortable ask for help at any time were found to be prospectively associated with decreased risks of both diagnosed depression and diagnosed anxiety (depression: RR = 0.794, 95% CI: 0.698, 0.904 and RR = 0.818, 95% CI: 0.731, 0.916, respectively; anxiety: RR = 0.847, 95% CI: 0.746, 0.962 and RR = 0.840, 95% CI: 0.743, 0.950, respectively, for each standard deviation of the exposure variable).

For variables “I feel connected to the broader community around me” and “People in my broader community trust and respect one another” there was a significant prospective association with reduced risks of clinical diagnosis of depression (RR = 0.809, 95% CI: 0.711, 0.919 and RR = 0.773, 95% CI: 0.676, 0.884, respectively). Additionally, these associations were also modestly robust to unmeasured confounding (Table 4), with associations with diagnosed depression being more robust than those with anxiety.

## Discussion

We found that social connectedness was prospectively associated with decreased risks of clinically diagnosed depression and clinically diagnosed anxiety. These associations were independent of demographics, socioeconomic status, lifestyle, work characteristics, and prior health conditions. They were also robust to unmeasured confounding, missing data patterns, extensive selection of control variables, and different model specifications. They applied to both, the subsample of people with no prior mental health conditions as well as to the entire analytical sample. We also showed that various aspects of social connectedness reflecting higher social connections, social support, and lack of feeling of isolation, were prospectively associated with decreased risks of clinically diagnosed depression and clinically diagnosed anxiety. However, for the perception of trust and respect among members of individual’s broader community as well as for the endorsement for feeling connected to one’s broader community, which reflect a collective form of social connectedness, we found that they were prospectively associated with reduced risk of clinically diagnosed depression but not with anxiety.

Findings of this study confirm related evidence from other studies that better social integration and social cohesion may provide benefits for mental health, while lack of them constitutes a health risk factor. Specifically, our results on the positive temporal association between a composite measure of social connectedness, experience of trust and respect in one’s broader community and lower odds of diagnosed depression corroborated prior evidence on the links between low neighborhood social cohesion and depression [43–45], as well as high neighborhood social cohesion and subsequent better psychological well-being [43] and better mental health measures [4]. Our findings on the importance of social support and social connections in particular, and on social connectedness in general, for reducing risks of depression and anxiety are in line with other studies reporting such associations. For example, previous evidence suggested that social integration was related to a decrease in depressive symptoms [46] and prevention of a depression relapse [7]. Our study also corroborated the meta-analytical results from 470 multidisciplinary both cross-sectional and longitudinal studies, indicating that various social factors, grouped under the umbrella term of social capital, are significantly related to numerous positive health outcomes [10]. However, our study reports much larger effect sizes, which could be related to our worker sample and/or to our use of objectively measured mental health outcomes. Further research is, however, required to find the reasons for these differences in effect sizes. Next, our findings on the prospective association of loneliness with increased risk of diagnosed depression and diagnosed anxiety, further corroborated earlier evidence on this link. In particular, a number of studies identified a link between loneliness and depression [11–14, 47], as well as between loneliness and anxiety [11, 15].

This study also provides some preliminary evidence on similar but also distinct roles of aspects of social connectedness that were focused on individual perspective and those reflecting a community perspective. Although Xue et al. [10], having meta-analyzed 12,778 estimates from 470 studies, reported that associations between health outcomes and social capital are robust across different types of social capital (e.g., cognitive, structural, bonding, bridging, linking), our results do not fully support their findings. Even though in our study the individually oriented indicators of social connectedness were associated with both subsequently diagnosed depression and anxiety, our community-oriented indicators of social connectedness were associated with subsequent diagnosis of depression only.

This study adds to the literature in the following ways. First, this study benefits from the data on diagnosed mental diseases that were derived from diagnostic information included in the medical insurance data and subsequently served as health outcomes. Prior studies have mostly used self-reported health information. Although it has been shown that self-reported health measures correspond with medical records, their accuracy and precision is not perfect [18, 48]. Thus, our findings provide stronger evidence in favor of prospective associations between social capital and mental health. Second, thanks to using the diagnostic information from health insurance claims (which classify diseases using the ICD-10), this study distinguished diagnosed depression from diagnosed anxiety and provided further evidence that these two diseases may be differently associated with prior community-related aspects of social connectedness. In this sense, our study corroborates prior findings on differential associations between depression and anxiety with worse psychosocial functioning (i.e., that higher levels of anxiety and depression correspond with worse psychosocial functioning but the effect of depression is progressively attenuated at higher levels of symptom severity [49]). We recognize that there is a substantial heterogeneity in qualifying for depression (including more than 280 ways to measure depression, and more than 50 distinct symptoms [50]) and practitioners are faced with challenges in discerning anxiety from depression. However, these two diseases are associated with distinct ICD-10 codes already provided by health practitioners in their diagnosis for our study participants. Fourth, the longitudinal design and the adjustment for a large set of covariates and the prebaseline outcomes as well as examination of alternative model specifications helped to establish temporal association and to strengthen evidence against reverse causation and unmeasured confounding. By using two perspectives to examine the associations between social connectedness and mental health, that is, community-related and general/individual-related, this study presents some patterns of associations that would not be noticeable if a single perspective had been applied. Finally, a series of secondary analyses and robustness analyses, including the sensitivity analysis for unmeasured confounding, provided reasonable evidence in favor of robustness of the prospective associations.

This study is subject to certain limitations. First, participants in this study were employees, mostly white collar workers, of a specific US employer, which limited generalizability of the results. Although the study design was planned to rely on random sampling, eventually only a subset of sampled employees provided data for the analyses. Second, attrition between waves 1 and 2 may be concerning. However, the comparison of basic demographic characterisitics of the participants and population of the organization provided evidence that these two groups are similar. Nevertheless, it would be advisable to replicate the results in different populations in the near future. Third, social connectedness was self-reported, so responses might have been subject to common methods bias and social desirability. Finally, we were not able to directly control for prebaseline social connectedness because of the reseach design that comprised two waves of survey data collection. However, we adjusted for a wide range of other potential confounders already established as contributors to mental health and health conditions. Additionally, we explicitly conducted sensitivity analysis for unmeasured confounding, which might substantially diminish concern about residual confounding.
