# Supplementary material for: Prospective Associations Between Social Connectedness and Mental Health. Evidence From a Longitudinal Survey and Health Insurance Claims Data
Source: Int J Public Health. 2022 Jun 9;67:1604710. doi: 10.3389/ijph.2022.1604710 (PMC9218058; doi:10.3389/ijph.2022.1604710)
Supplement: Supplementary file 1 [file DataSheet1.PDF]

Prospective associations between social connectedness and mental health. Evidence from a longitudinal survey and health insurance claims data.

International Journal of Public Health

Supplementary Materials

Table A1. Prevalence of missing data (Well-Being Survey 2018-2019 and health insurance claims data 2017-2019, N=1,209).

**Variables**	% of missing observations
Control variables	
Gender	0.0
Age	0.0
Race	0.0
Marital status	1.49
Education	1.08
Having children under the age of 18 currently living in the household	1.32
Being a primary caregiver for a parent or an elderly currently living in the household	1.08
Home ownership	1.24
Salary (USD)	0.0
Voting in the previous elections	1.08
Spiritual practicing	0.99
Work hours	1.08
I have too much to do at work to do a good job	1.65
I have a lot of freedom to decide how to do my job	1.99
I find my work meaningful	1.49
At work, I am able to do what I am good at	1.65
My supervisor supports me	1.32
Number of health conditions	0.0
Independent variables	
Social connectedness	3.64
My relationships are as satisfying as I would want them to be	1.08
There are people who really understand me	0.83
How often do you feel lonely?	0.99
I am content with my friendships and relationships	0.99
I have enough people I feel comfortable asking for help at any time	1.32
I feel connected to the broader community around me	1.41
People in my broader community trust and respect one another	1.16
Mental health outcomes from health insurance records	
Depression	0.0
Anxiety	0.0

Characteristics of study participants

In the baseline wave (T=0), participants were 43.5 (SD = 10.4) years old, on average (Table 1). They were mostly women (84.5%), married (62.5%), predominantly Caucasian (74.3), and with Bachelor's degree (35.0%). More than 72% of the participants reported owning a home, 82.4% participated in the previous elections and 91.8% declared to engage in spiritual practices. Regarding working conditions, 52.5% of the participants reported working at least 8h per day, while 35.4% reported 9–10h. Their reports regarding job control, job demand, supervisor's support, meaning of work and job fit were well above the middle response category. They were also relatively healthy (mean number of health conditions amounted to 2.0 with the theoretical maximum of 37 conditions).

With respect to mental health in particular, in the baseline wave (T=0) 10.4% of the participants were diagnosed with depression and 12.2% were diagnosed with anxiety (Table 2). However, no significant changes in anxiety were noted between the prebaseline (2017) and 2019 but prevalence of depression increased over this period.

Regarding social connectedness, in the baseline wave (T=0, 2018), participants scored 7.4 on average on a 0-10 response scale [Table 2, (30)]. Regarding specific indicators of social connectedness, the average scores were between 6.7 (feeling connected to the broader community around me) and 7.9 (there are people who really understand me) for the positively oriented items, and 2.3 for the indicator of loneliness. Additionally, these scores improved between 2018 and 2019 (T=0 and T=1). Specifically, in 2019, in comparison to 2018, participants on average reported higher scores in the composite social connectedness score and in all measured aspects of social connectedness (with the exception of feeling lonely question, for which p-value was 0.012, in all

remaining cases for one sided t-test for paired observations the p-value<0.001 was reported) (p<0.001).

Table A2. Reciprocal associations between social connectedness and subsequent mental health (Well-Being Survey 2018-2019 and health insurance claims data 2017-2019) – cross-lagged panel model.

**Depression**			**Anxiety**	
	**Path estimate 95% CI**			**Path estimate 95% CI**
	Depression 2019			Anxiety 2019
Social connectedness 2018	-0.403** (-0.682, -0.123)		Social connectedness 2018	-0.309* (-0.552, -0.065)
Depression 2018	5.544*** (4.675, 6.374)		Anxiety 2018	3.902*** (3.344, 4.459)
	Social connectedness 2019			Social connectedness 2019
Social connectedness 2018	0.646*** (0.600, 0.692)		Social connectedness 2018	0.656*** (-0.342, 0.139)
Depression 2018	-0.187* (-0.330, -0.044)		Anxiety 2018	-0.069 (-0.201, 0.063)

Each analysis was controlled for demographics (gender, age, race, education, marital status, having children at home, taking care of an elderly), wealth and income (home ownership and salary), lifestyle (voting in the last elections and spiritual practices) and work characteristics (number of work hours, supervisor support, job control, job demand and job meaning). These variables were controlled for in the first wave (in the same wave as the exposure), since only two waves of survey data were available. Additionally, in each regression an outcome prior to exposure as well as the number of diagnosed health conditions (ranging from 0 to 37 possible diagnosed health conditions) prior to exposure were applied as controls.

Social connectedness 2018 and social connectedness 2019 were standardized.

Not imputed sample was used because in Stata *gsem* does not work with *mi estimate*.

Supplementary Analyses

Alternative model specifications were tested to provide additional evidence on the robustness of the examined associations between social connectedness and mental health outcomes. First, a between-effect model in which a 2019-2018 difference in mental health outcome is regressed on a 2019-2018 difference in social connectedness exposure (controlling for the set of covariates from the primary analysis and pre-exposure history of diseases) was estimated (Model S1). Second, a model with two instantaneous effects was estimated: (i) mental health outcome in 2018 was regressed on social connectedness in 2018 and mental health outcome in 2019 was regressed on social connectedness in 2019 (controlling for the set of covariates from the primary analysis and pre-exposure history of diseases; Model S2). Third, three lag-effect models were also examined. In Model S3.1 a mental health outcome in T=1 was regressed on social connectedness in T=0, controlling for the set of covariates from the primary analysis and pre-exposure history of diseases as well as social connectedness in T=1. In Model S3.2 a mental health outcome in T=1 was regressed on a mental health outcome in T=0, controlling for the set of covariates from the primary analysis and pre-exposure history of diseases as well as social connectedness in T=1. In Model S3.3, a mental health outcome in T=1 was regressed on both on social connectedness in T=0 and a mental health outcome in T=0, controlling for the set of covariates from the primary analysis and pre-exposure history of diseases as well as social connectedness in T=1.

Table A3. Reciprocal associations between social connectedness and subsequent mental health – alternative model specifications (Well-Being Survey 2018-2019 and health insurance claims data 2017-2019)[a].

Model specification	Depression		Anxiety	
	**OR 95% CI**		**OR 95% CI**	
Model S1[b]	1.013 (0.667, 1.539)		1.056 (0.723, 1.543)	
Model S2[c]	T=0	0.587** (0.406, 0.796)	T=0	0.837 (0.633, 1.106)
	T=1	0.623*** (0.491, 0.790)	T=1	0.740** (0.602, 0.910)
Model S3.1[d]	0.634* (0.505, 0.954)		0.816 (0.616, 1.081)	
Model S3.2[e]	135.711*** (50.683, 363.388)		34.206*** (16.825, 69.541)	
Model S3.3[f]	Social connectedness T=0	0.815 (0.558, 1.189)	Social connectedness T=0	0.767 (0.555, 1.060)
	Depression T=0	127.391*** (47.493, 341.699)	Anxiety T=0	34.161*** (16.678, 69.969)

***p<0.001, **p<0.01, *p<0.05; CI is confidence interval.

CI=confidence interval; OR=odds ratio

[a] Each analysis was controlled for demographics [gender (ref.=female), age (ref.=below 30), race (ref.=White), education (ref.=high school), marital status (ref.=not married)), having children at home (ref.=no), taking care of an elderly (ref.=no)], wealth and income [home ownership (ref.=no) and salary], lifestyle [voting in the last elections (ref.= no/not registered voter) and spiritual practices (ref.=at least once/week)] and work characteristics (number of work hours (ref.= 8h and less than 8h), supervisor support, job control, job demand and job meaning). These variables were controlled for in the baseline wave, T=0 (in the same wave as the exposure), since only two waves of survey data were available. Additionally, in each regression an outcome prior to exposure (T=-1) as well as the number of diagnosed health conditions (ranging from 0 to 37 possible diagnosed health conditions) prior to exposure (T=-1) were used as controls. All models are run on non-imputed dataset to keep all models consistent in terms of estimation approach as some function is Stata (e.g., *gsem*) do not work with *mi estimate*.

[b] Model S1 is a between-effect model in which a 2019-2018 difference in mental health outcome is regressed on a 2019-2018 difference in social connectedness exposure. This model is run the sample of adults who did not suffer from depression/anxiety in 2018.

[c] Model S2 is a model with two instantaneous effects: (i) mental health outcome in 2018 was regressed on social connectedness in 2018 and mental health outcome in 2019 was regressed on social connectedness in 2019.

[d] Model S3 is a lag-effect model in which a mental health outcome in T=1 was regressed on social connectedness in T=0, controlling for the set of covariates from the primary analysis and pre-exposure history of diseases as well as social connectedness in T=1.

[e] In Model S3.2 a mental health outcome in T=1 was regressed on a mental health outcome in T=0, controlling for the set of covariates from the primary analysis and pre-exposure history of diseases as well as social connectedness in T=1.

[f] In Model S3.3, a mental health outcome in T=1 was regressed on both on social connectedness in T=0 and a mental health outcome in T=0, controlling for the set of covariates from the primary analysis and pre-exposure history of diseases as well as social connectedness in T=1.

Results from the secondary analyses which tested alternative specifications showed that there are no concurrent associations between differences in the mental health outcome and social connectedness for both depression and anxiety (Supplementary Materials, Table A3, Model S1) but there is a concurrent association between social connectedness and depression in both T=0 and T=1. However, for anxiety such association is observed only in T=1 but not in T=0 (Supplementary Materials, Table A3, Model S2). Finally, three lag-effect models showed that (1) social connectedness in T=0 was prospectively associated with depression in T=1 but not with anxiety; (2) social connectedness in T=0 was associated with subsequent depression (in T=1) and with subsequent anxiety (in T=1); (3) social connectedness in T=0 was not prospectively associated with either depression in T=1 or anxiety in T=1, however each mental health outcome in T=0 was found to be associated with respective health outcome in T=1 (Supplementary Materials, Table A3, Model S3.1-S3.3). These analyses provided further evidence on the robustness of prospective associations. The evidence on causal relationships was very limited, which supported arguments that observational data are substantially imperfect to interpret the results in causal terms. To this end, data from scientific experiments relying on randomized controlled trials are more useful.
